# Stage-dependent performance and molecular subtype-specific relapse patterns on same-session [18F]FDG PET/contrast-enhanced CT in breast cancer

**DOI:** 10.1007/s00259-026-08077-w

**Published:** 2026-08-07

**Authors:** Agostino Chiaravalloti, Shyqyri Samarxhiu, Ilaria Portarena, Valeria Flaminio, Nicola D’Ascenzo, Orazio Schillaci, Gianluca Vanni

**Affiliations:** 1 Department of Biomedicine and Prevention, University of Rome Tor Vergata, Viale Montpellier 1, Rome, 00133 Italy; 2 IRCCS Neuromed, Via Atinense 18, Pozzilli, 86077 IS Italy; 3 Medical Oncology Unit, Fondazione PTV Policlinico Tor Vergata, Viale Oxford 81, Rome, 00133 Italy; 4 Department of Electronic Engineering and Information Science, University of Science and Technology of China, Hefei, Anhui China; 5 Department of Biomedical Engineering, Huazhong University of Science and Technology, Wuhan, Hubei China; 6 Breast Unit, Department of Surgical Sciences, Fondazione PTV Policlinico Tor Vergata, University of Rome Tor Vergata, Rome, 00133 Italy

**Keywords:** Breast cancer, [18F]FDG PET/CT, Contrast-enhanced CT, Staging, Restaging, Molecular subtype

## Abstract

**Purpose:**

To evaluate stage-dependent performance of same-session [18F]FDG PET/contrast-enhanced CT (PET/ceCT) for initial breast cancer staging and PET/ceCT-detected relapse patterns during follow-up.

**Methods:**

This retrospective single-centre study included a primary staging cohort with one index PET/ceCT per patient and a longitudinal cohort of follow-up/restaging examinations. PET/ceCT-derived endpoints were assessed against clinically adjudicated reference standards established through multidisciplinary team review, pathology when available and follow-up imaging. SUVmax and ceCT lesion diameter were analysed as exploratory biomarkers.

**Results:**

The staging cohort included 116 patients. Baseline stage was evaluable in 106 patients: 65 had cN-positive disease, 9 had cT3-4 cN0 disease and 32 had cT1-2 cN0 disease. Overall nodal staging performance showed sensitivity 64.8%, specificity 88.9%, PPV 92.0%, NPV 56.1% and accuracy 72.9%. In cT3-4 and/or cN-positive disease, PET-positive nodal findings had a PPV of 100%. Baseline M-stage assessment was limited by the low number of clinically confirmed M1 cases. SUVmax correlated with Ki-67 (rho = 0.322, *p* = 0.003, q = 0.022). The longitudinal cohort included 176 patients and 429 examinations. In 311 adjudicated examinations, restaging performance for disease progression showed sensitivity 100%, specificity 93.5%, PPV 97.3%, NPV 100% and accuracy 98.1%. First confirmed relapse was multiorgan/multisystem in 48/82 patients and visceral-only in 15/82; visceral conversion occurred in 5/16 patients with initial bone-only or nodal/locoregional relapse.

**Conclusion:**

Same-session [18F]FDG PET/ceCT showed greatest baseline utility in higher-risk breast cancer and was useful for longitudinal characterization of relapse phenotype and metastatic evolution.

**Supplementary Information:**

The online version contains supplementary material available at https://doi.org/10.1007/s00259-026-08077-w.

## Introduction

Breast cancer is a biologically heterogeneous disease in which treatment allocation depends on the integration of anatomical stage, histology, tumour grade, hormone receptor status, HER2 expression, proliferative activity and clinical risk. Contemporary guidelines recommend that pathological assessment at diagnosis should include histological type, grade, ER, PR, HER2 and a proliferation marker such as Ki-67, and that tumours should be grouped into biological subtypes based on routine histology and immunohistochemistry [1]. Accurate baseline staging remains essential because detection of extra-locoregional nodal disease or distant metastases may substantially modify treatment intent, radiotherapy planning and systemic treatment strategy.

The use of systemic imaging in newly diagnosed breast cancer is risk-adapted rather than universal. ESMO guidelines recommend assessment of distant metastases mainly in patients with stage IIb or higher disease, extended nodal involvement, high-risk disease at diagnosis, symptoms, or laboratory abnormalities suggestive of metastatic spread; FDG PET/CT may be used instead of CT and bone scintigraphy, particularly in high-risk patients or when conventional methods are inconclusive [1]. The recent joint EANM-SNMMI guideline further refines this position, stating that 2-[18F]FDG PET/CT has a role in baseline staging from stage IIB through stage IV, is not recommended in stage I, and may be useful in selected stage IIA cases [2]. This stage-dependent framework is clinically important, because the diagnostic yield of PET/CT is expected to vary according to pre-test probability.

Recent evidence supports this selective approach. In a systematic review and meta-analysis including 29 studies and 4276 patients, FDG PET, PET/CT or PET/MRI changed initial stage in 25% of patients and management in 18%; stage modification increased with baseline stage, from 11% in stage I to 20% in stage II and 34% in stage III [3]. In a prospective study of high-risk primary breast cancer, FDG PET/CT identified distant metastases in 23% of patients, upstaged another 16% to more advanced locoregional disease, and showed sensitivity and specificity of 100% and 95% for distant metastasis using biopsy and follow-up as the reference standard [4]. In locally advanced breast cancer, PET/CT has also been shown to detect more nodal and distant disease than conventional imaging, with stage migration and management changes in a substantial proportion of patients [5]. These data support a central clinical question: not whether PET/CT should be used indiscriminately, but in which baseline clinical stages and biological subgroups it is most informative.

Nodal staging remains a particularly relevant area for PET/CT evaluation. Although PET/CT cannot replace surgical axillary staging in early breast cancer, it may identify clinically relevant axillary, internal mammary, supraclavicular or other extra-axillary nodal disease that can influence treatment planning. Davidson et al. reported FDG-avid axillary lymphadenopathy in 48.1% of newly diagnosed breast cancer patients, with metastases confirmed in 85.5% of PET-positive axillary nodes that underwent further assessment; distant FDG-avid lesions were detected in 10.2% of patients [6]. These findings are consistent with a high positive predictive value of PET/CT-positive nodal findings, while also highlighting that PET-negative axilla cannot reliably exclude microscopic disease.

Same-session PET/contrast-enhanced CT (PET/ceCT) is particularly relevant because it integrates metabolic and diagnostic morphologic information in a single examination. The EANM-SNMMI guideline states that concurrent contrast-enhanced CT may be considered to improve lesion detection and to facilitate comparison with follow-up CT, especially when evaluating treatment response in metastatic disease [2]. However, the added value of the contrast-enhanced CT component should be interpreted cautiously unless PET-only and PET/ceCT readings are compared in a dedicated design. In a retrospective clinical cohort, PET/ceCT is more appropriately evaluated as an integrated hybrid examination, with discordant PET and ceCT findings described as an exploratory secondary endpoint.

Beyond lesion detection, FDG uptake reflects tumour biology. A recent systematic review and meta-analysis including 50 studies showed that primary tumour SUVmax is higher in ER-negative, PR-negative, HER2-positive and Ki-67-positive breast cancer, with luminal tumours showing the lowest and triple-negative tumours the highest FDG uptake [7]. The EANM-SNMMI guideline similarly emphasizes that FDG avidity is related to histologic type, receptor status, grade, Ki-67 and tumour size, and that quantitative PET features such as SUV, metabolic tumour volume and total lesion glycolysis are valuable prognostic imaging biomarkers [2]. These observations justify correlating SUVmax and ceCT-measured index lesion diameter with histology, grade, ER, PR, Ki-67, HER2 status, molecular subtype, clinical stage, radiological response and pathological outcome.

FDG PET/CT may also provide useful information in the neoadjuvant setting. In triple-negative breast cancer, pretreatment PET-derived metrics such as SUVmax and metabolic tumour volume have been associated with pathological complete response after neoadjuvant chemotherapy with or without pembrolizumab [8]. Similarly, early SUVmax reduction during neoadjuvant treatment has been reported as a strong predictor of pathological complete response across breast cancer subtypes [9]. Although these studies address response prediction rather than baseline staging accuracy, they reinforce the biological relevance of PET-derived quantitative parameters and support their inclusion as exploratory correlates in clinically annotated PET/ceCT cohorts.

In restaging and follow-up, PET/CT addresses a different clinical question. Rather than simply assigning baseline stage, serial PET/CT can characterize the pattern and evolution of relapse, including bone-only, visceral-only, nodal/locoregional and multiorgan disease. In metastatic breast cancer, FDG PET/CT has shown high value for response evaluation, with metabolic changes often preceding morphologic progression on conventional CT [10]. In a prospective study comparing FDG PET/CT with contrast-enhanced CT for response monitoring in metastatic breast cancer, progression was detected first by PET/CT in 49.4% of patients and first by contrast-enhanced CT in only 1.15%, with a median 6-month interval between PET/CT detection and subsequent CT detection of progression [11]. Importantly, that cohort included a high proportion of patients with bone metastases, and the reported advantage of PET/CT was partly related to metabolic assessment of bone-dominant disease, a setting in which morphologic CT response assessment is intrinsically limited. These findings support the use of longitudinal PET/CT not only for detecting relapse, but also for defining metastatic evolution over time.

Most available studies have focused either on baseline staging or on metastatic response assessment. Fewer have combined a clinically adjudicated analysis of stage-dependent PET/ceCT performance at initial staging with longitudinal evaluation of relapse phenotypes according to molecular subtype. This distinction is important: at baseline, the clinically relevant question is when PET/ceCT provides reliable staging information; during follow-up, the relevant question is how PET/ceCT captures relapse pattern, time to distant relapse and conversion to visceral or multiorgan disease.

The aim of this study was therefore to evaluate the stage-dependent performance of same-session [18F]FDG PET/ceCT in breast cancer patients undergoing initial staging, using clinically confirmed reference standards derived from pathology, follow-up imaging, treatment decisions and standard-of-care clinical assessment. Secondary aims were to assess molecular subtype-specific patterns of PET/ceCT-detected distant relapse during longitudinal follow-up, and to explore associations between SUVmax, ceCT-measured index lesion diameter, clinicopathological variables, response parameters and discordant PET/ceCT findings.

## Materials and methods

### Study design and population

This retrospective single-centre cohort study included patients with histologically confirmed breast cancer who underwent same-session [18F]FDG PET/contrast-enhanced CT (PET/ceCT) as part of standard clinical care. Clinical, pathological, imaging and follow-up data were reviewed from institutional clinical, pathology and imaging records.

**Fig. 1:**
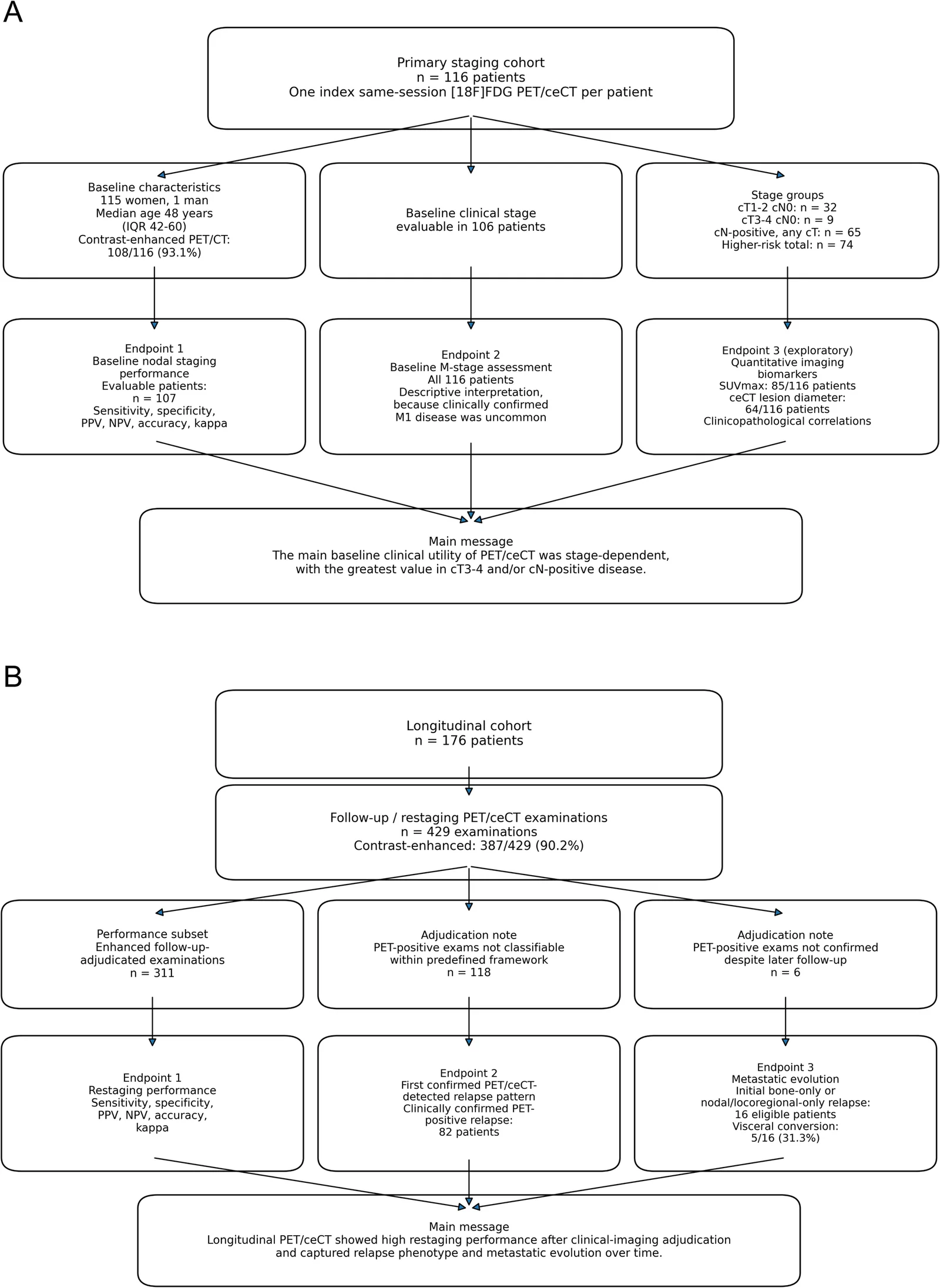
**A**. Primary staging cohort. Flow chart summarizing the primary staging cohort. The cohort included 116 patients with one index same-session [18F]FDG PET/ceCT examination per patient. The baseline clinical-stage breakdown included 32 patients with cT1-2 cN0 disease, 9 with cT3-4 cN0 disease and 65 with cN-positive disease irrespective of cT category. The main baseline endpoints were nodal staging performance, descriptive M-stage assessment and exploratory quantitative correlations between imaging metrics and clinicopathological variables. Abbreviations: PET/ceCT, same-session [18F]FDG PET/contrast-enhanced CT; PPV, positive predictive value; NPV, negative predictive value. **B**. Longitudinal cohort. Flow chart summarizing the longitudinal cohort. The cohort included 176 patients and 429 follow-up/restaging PET/ceCT examinations, of which 387/429 (90.2%) included contrast enhancement. Restaging performance for disease progression was assessed in the enhanced follow-up-adjudicated subset (*n* = 311 examinations). Additional endpoints were first confirmed PET/ceCT-detected relapse pattern and metastatic evolution over time. PET-positive examinations outside the adjudicated diagnostic-performance subset were excluded from specificity calculations

Two analytically distinct cohorts were defined. The primary staging cohort included patients who underwent PET/ceCT for initial staging, according to predefined clinical matching criteria and with only one index examination per patient. This cohort was used to evaluate stage-dependent PET/ceCT performance, with a primary focus on nodal involvement and distant metastatic disease at baseline. The longitudinal cohort included patients who underwent subsequent PET/ceCT examinations during follow-up, restaging or suspected recurrence. This cohort was used to assess PET/ceCT-detected relapse patterns, time to relapse, metastatic evolution and restaging performance using longitudinal clinical-imaging adjudication. The structure of the primary staging cohort and longitudinal cohort, together with their respective endpoints, is summarized in Fig. 1A-B.

Duplicate examinations, examinations without reliable clinical matching, cases in which PET/ceCT indication could not be assigned to initial staging or follow-up/restaging, and lesions finally classified as second primary malignancies rather than breast cancer metastases were excluded from disease-specific performance analyses.

### Clinical and pathological variables

The following variables were collected when available: sex, age at PET/ceCT, histological type, tumour grade, ER status, PR status, Ki-67, HER2 score and HER2 positive/negative status, hormone receptor status, molecular subtype, baseline cT and cN stage, interim cT/cN stage after 3–4 treatment cycles, final ycT/ycN stage, radiological response, background parenchymal enhancement, pathological complete response, final histology, ypT, ypN, tumour size, ductal carcinoma in situ component, comedonecrosis, microinvasion, T-category components, grading, lymphovascular invasion and adjuvant/neoadjuvant treatment variables.

Molecular subtypes were assigned according to routine pathological classification based on ER, PR, HER2 and Ki-67, and grouped for analysis as luminal A, luminal B/HER2-negative, luminal B/HER2-positive, HER2-type and basal-like/triple-negative. For selected analyses, molecular subtypes were also collapsed into clinically robust categories: hormone receptor-positive/HER2-negative, HER2-positive and triple-negative/basal-like disease.

### PET/ceCT acquisition and image assessment

All PET/ceCT examinations were performed according to institutional protocols, as previously described [12], with technical adaptations over the study period. Examinations performed before 2014 were acquired on a GE Discovery VCT PET/CT system, whereas examinations performed after 2020 were acquired on a GE Discovery MI PET/CT system. Examinations performed in the intermediate period were acquired on the GE PET/CT systems available at our institution according to routine clinical protocols.

Patients fasted for at least 5 h before intravenous [18F]FDG administration, and blood glucose levels were checked before tracer injection according to routine clinical practice. Physical activity was minimized during the uptake phase, and image acquisition started approximately 60 min after [18F]FDG injection.

PET/CT was acquired from the upper thighs to the vertex. A CT scan was first obtained for attenuation correction and anatomical localization, followed by whole-body PET acquisition. PET data were reconstructed according to the acquisition and reconstruction standards available at the time of scanning. When clinically indicated and technically feasible, diagnostic contrast-enhanced CT was acquired during the same examination session after intravenous administration of iodinated contrast medium and interpreted together with PET as an integrated PET/ceCT examination.

PET/ceCT images were retrospectively reviewed on dedicated workstations allowing separate and fused visualization of PET and CT datasets in axial, coronal and sagittal planes. Imaging findings were assessed as integrated metabolic-morphological findings, rather than as isolated PET-only or CT-only abnormalities. Focal [18F]FDG uptake higher than surrounding background and not attributable to physiological distribution or benign processes was considered suspicious for disease involvement. No absolute SUVmax threshold was used for lesion classification. In equivocal lesions, the co-registered contrast-enhanced CT component and subsequent clinical or imaging follow-up were used to support final adjudication.

For the staging cohort, PET/ceCT-derived nodal status, M stage, lesion site, SUVmax and maximum lesion diameter on ceCT were recorded. For the longitudinal cohort, serial PET/ceCT examinations were reviewed to identify first confirmed relapse, relapse pattern, disease progression, response or disappearance of previously described lesions. Metastatic lesions were distinguished from common benign pitfalls, including fractures, degenerative changes, inflammatory findings and post-treatment changes, using the integrated PET/ceCT appearance and follow-up information when available.

Because PET/ceCT examinations spanned different scanner generations and reconstruction protocols, semiquantitative analyses based on SUVmax were considered exploratory and were interpreted in conjunction with lesion site, tumour size, histology, molecular subtype and clinical context.

### Reference standard and endpoint definitions

The reference standard was clinically adjudicated using pathology when available, follow-up imaging, multidisciplinary team review or clinical assessment, treatment decisions and clinical evolution. For baseline staging, PET/ceCT-derived nodal status and M stage were compared with the final clinically confirmed nodal and metastatic status. Baseline M1 disease required clinical confirmation of distant metastatic breast cancer. Lesions ultimately classified as second primary malignancies were not counted as breast cancer M1 disease.

For follow-up/restaging analyses, PET/ceCT-positive relapse or progression was considered confirmed by clinical-imaging adjudication when supported by at least one of the following: concordant clinical documentation; histology or biopsy when available; progression at the same site or disease category on subsequent imaging; persistence or stability of suspicious lesions on follow-up in a clinically consistent context; or disappearance or marked reduction of lesions after treatment, consistent with response of true disease. In this manuscript, “confirmed relapse” refers to this predefined clinical-imaging adjudication framework and does not imply histological confirmation in every case. PET-positive findings not classifiable within the predefined adjudication framework were not included in diagnostic performance calculations.

The primary endpoint was the stage-dependent performance of same-session PET/ceCT in the initial staging cohort, using clinically confirmed reference standards. Diagnostic performance was evaluated for nodal involvement and M-stage classification. Analyses were performed overall and stratified by baseline clinical risk, particularly cT1-2 cN0 versus cT3-4 and/or cN-positive disease.

Secondary endpoints in the staging cohort included PET/ceCT performance according to clinical stage, PET/ceCT-positive nodal predictive value in higher-risk disease, detection of baseline distant disease, SUVmax and ceCT-measured index lesion diameter according to molecular subtype and clinicopathological variables, association of PET/ceCT findings with pCR, ypN status and response variables, and exploratory analysis of discordant PET versus contrast-enhanced CT findings.

The major longitudinal endpoint was the pattern of first confirmed PET/ceCT-detected relapse according to molecular subtype. Relapse patterns were classified as bone-only, visceral-only, nodal/locoregional-only, multiorgan or multisystem, other-only, or no confirmed PET/ceCT-detected relapse. Visceral relapse included lung, liver, brain, adrenal, peritoneal or other solid-organ involvement. Multiorgan/multisystem disease was defined as involvement of more than one anatomical disease compartment. Oligometastatic disease was defined as a limited number of countable metastatic lesions, whereas polymetastatic disease included multiple, diffuse or non-countable metastatic lesions. When lesion count was not reliable, the oligometastatic/polymetastatic classification was not forced.

Time to PET/ceCT-detected relapse was calculated from surgery or initial treatment date to the first confirmed PET/ceCT-positive relapse. Conversion to visceral disease was defined as subsequent development of visceral or multiorgan disease after an initial bone-only or nodal/locoregional-only relapse pattern.

### PET versus contrast-enhanced CT discordance

Discordant PET/ceCT findings were analysed as an exploratory secondary endpoint and classified as concordant PET-positive and ceCT-positive, PET-positive without clear CT correlate, ceCT-positive or morphologically suspicious without significant FDG uptake, mixed discordance, or no relevant PET/ceCT discordance.

This analysis was not designed to formally quantify the incremental diagnostic value of contrast-enhanced CT over PET alone, because PET-only and PET/ceCT readings were not performed as an independent blinded reader-study. Instead, it was used to describe the clinical contribution of integrated metabolic and morphologic assessment.

### Statistical analysis

Categorical variables were reported as counts and percentages. Continuous variables were reported as median and interquartile range or mean and standard deviation according to distribution. Normality was assessed using the Shapiro-Wilk test.

Diagnostic performance of PET/ceCT was calculated using the clinically adjudicated reference standard. Sensitivity, specificity, positive predictive value, negative predictive value and accuracy were reported with 95% confidence intervals when appropriate. Agreement between PET/ceCT-derived classification and reference standard was assessed using Cohen’s kappa.

Comparisons between categorical variables were performed using the chi-square test or Fisher’s exact test, as appropriate. Continuous variables were compared using the Mann-Whitney U test or Kruskal-Wallis test for non-normally distributed data, and Student’s t test or analysis of variance for normally distributed data. Associations between SUVmax, ceCT-measured index lesion diameter and continuous clinicopathological variables were assessed using Spearman’s rank correlation.

Exploratory correlation analyses included SUVmax and ceCT-measured index lesion diameter versus histology, tumour grade, ER, PR, Ki-67, HER2, hormone receptor status, molecular subtype, cT/cN stage, response parameters, pCR, ypT, ypN, lymphovascular invasion and treatment variables. To control for multiple testing in exploratory analyses, p values were adjusted using the Benjamini-Hochberg false discovery rate procedure, and q values were reported when appropriate. The q value was defined as the Benjamini-Hochberg false discovery rate-adjusted p value, representing the minimum false discovery rate at which a given association would be considered significant.

Stage-dependent performance analyses were performed by stratifying the staging cohort according to baseline clinical stage, particularly cT1-2 cN0 versus cT3-4 and/or cN-positive disease. Longitudinal analyses were performed at patient level for first confirmed relapse pattern and at examination level for restaging performance using longitudinal clinical-imaging adjudication.

A two-sided p value < 0.05 was considered statistically significant. Exploratory analyses were interpreted cautiously, with emphasis on effect size, biological plausibility and false discovery rate-adjusted significance.

## Results

### Study population

The primary staging cohort included 116 patients with histologically confirmed breast cancer who underwent one index same-session [18F]FDG PET/ceCT examination for initial staging. The cohort included 115 women and 1 man, with a median age of 48 years (IQR 42–60). Intravenous contrast-enhanced CT was performed in 108/116 patients (93.1%).

Baseline clinical stage was evaluable in 106 patients. Of these, 32/106 (30.2%) had cT1-2 cN0 disease, 9/106 (8.5%) had cT3-4 cN0 disease, and 65/106 (61.3%) had cN-positive disease irrespective of cT category. The latter two categories constituted the higher-risk subgroup of 74/106 patients (69.8%), defined as cT3-4 and/or cN-positive disease. Molecular subtype distribution, histology, grading, receptor profile, Ki-67, clinical stage, response parameters and pathological outcomes are summarized in Table 1 and Supplementary Table 1.

**Table 1:** Baseline characteristics of the primary staging cohort

Variable	Result	Variable	Result
Patients	116	Women / men	115 / 1
Age, years	48, IQR 42–60	Same-session PET/ceCT with intravenous contrast	108/116, 93.1%
Baseline clinical stage evaluable	106/116	cT1-2 cN0	32/106, 30.2%
cT3-4 and/or cN+	74/106, 69.8%	SUVmax available for quantitative/subtype analysis	85/116, 73.3%
ceCT-measured index lesion diameter available	64/116, 55.2%	Histology evaluable	108
Invasive ductal carcinoma / NST	94/108, 87.0%	Invasive lobular carcinoma	2/108, 1.9%
Other / special / not otherwise specified	12/108, 11.1%	Tumour grade evaluable	39
G1	5/39, 12.8%	G2	17/39, 43.6%
G3	17/39, 43.6%	ER evaluable	105
ER-positive	80/105, 76.2%	ER-negative	25/105, 23.8%
PR evaluable	105	PR-positive	69/105, 65.7%
PR-negative	36/105, 34.3%	HER2 evaluable	105
HER2-positive	33/105, 31.4%	HER2-negative	72/105, 68.6%
Ki-67 evaluable	105	Ki-67, median, IQR	35%, IQR 20–55
Molecular subtype evaluable	113	HR+/HER2-	59/113, 52.2%
HER2-positive overall	33/113, 29.2%	Triple-negative/basal-like	18/113, 15.9%
Other / not classifiable	3/113, 2.7%	cT evaluable	95
cT1	10/95, 10.5%	cT2	61/95, 64.2%
cT3	13/95, 13.7%	cT4	11/95, 11.6%
cN evaluable	96	cN0	31/96, 32.3%
cN1	52/96, 54.2%	cN2	11/96, 11.5%
cN3	2/96, 2.1%	pCR evaluable	90
pCR	35/90, 38.9%	Non-pCR	55/90, 61.1%
ypN evaluable	90	ypN-positive residual disease	34/90, 37.8%
ypN0	56/90, 62.2%		
cT3-4 cN0	9/106, 8.5%	cN-positive, any cT category	65/106, 61.3%
Higher-risk group overall	74/106, 69.8%		

A separate longitudinal cohort included 176 patients who underwent PET/ceCT during follow-up, restaging or suspected recurrence. Overall, 429 PET/ceCT examinations were evaluated, of which 387/429 (90.2%) included contrast-enhanced CT. Serial PET/ceCT follow-up was available in a substantial subset, allowing assessment of first confirmed PET/ceCT-detected relapse, relapse phenotype and metastatic evolution over time. The study workflow and endpoint structure for the primary staging and longitudinal cohorts are shown in Fig. 1A-B.

### PET/ceCT performance in initial staging

**Fig. 2:**
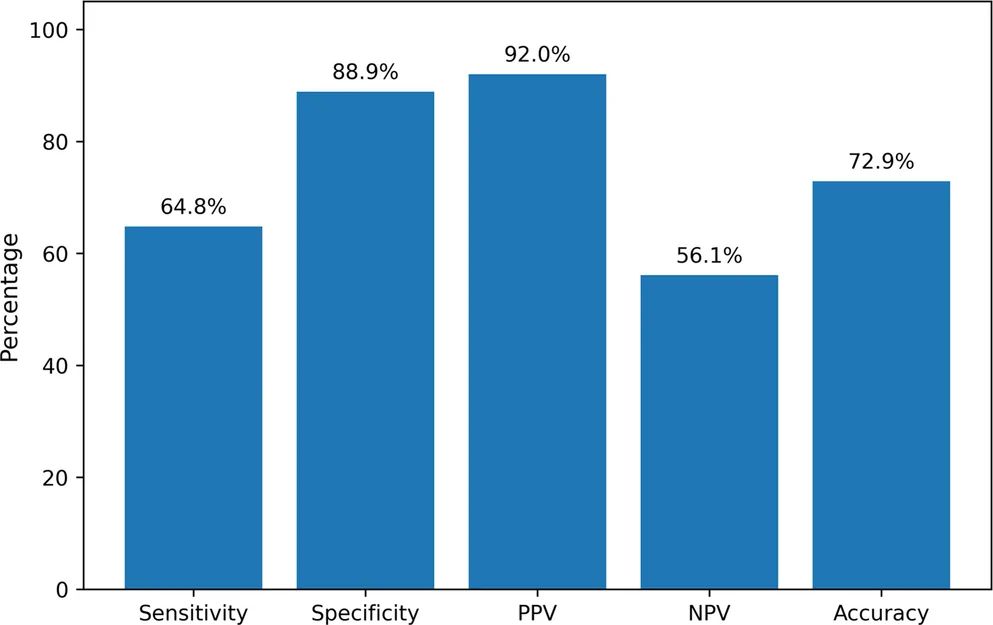
Baseline nodal staging performance. Bar chart showing the main performance metrics of PET/ceCT for baseline nodal staging in 107 evaluable patients. Sensitivity was 64.8%, specificity 88.9%, PPV 92.0%, NPV 56.1%, and accuracy 72.9%. TP = 46, FP = 4, TN = 32, and FN = 25. Cohen’s kappa was 0.469

Nodal staging was evaluable in 107 patients. Overall, PET/ceCT showed sensitivity 64.8%, specificity 88.9%, PPV 92.0%, NPV 56.1%, accuracy 72.9% and Cohen’s kappa 0.469 for clinically confirmed nodal involvement. Overall baseline nodal staging performance is shown in Fig. 2.

**Fig. 3:**
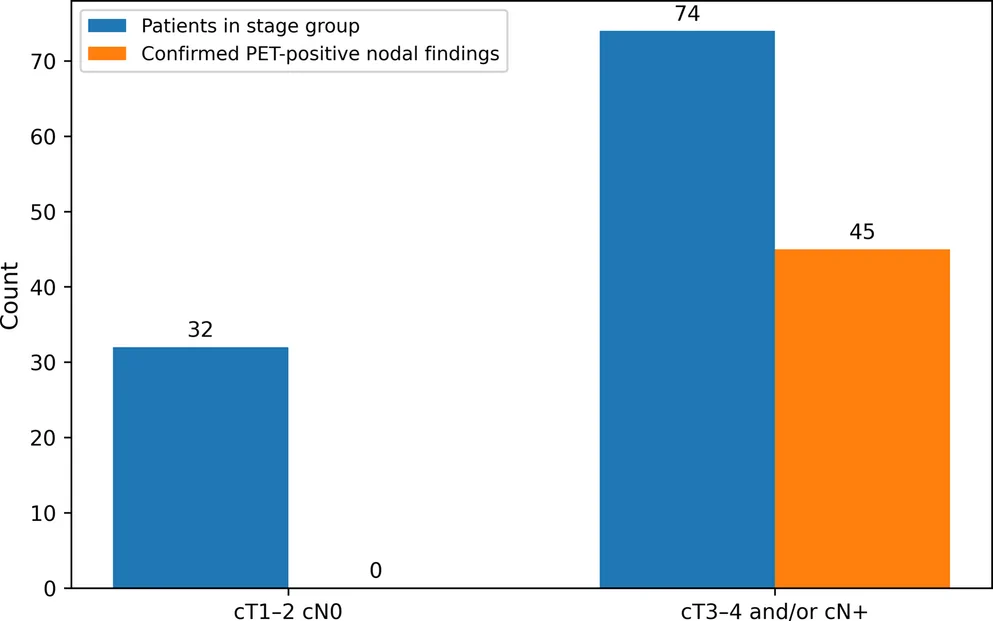
Stage-dependent baseline clinical yield. Graph illustrating the stage-dependent utility of nodal PET/ceCT. Baseline clinical stage was evaluable in 106 patients, including 32 with cT1-2 cN0 disease and 74 in the higher-risk subgroup (9 with cT3-4 cN0 disease and 65 with cN-positive disease irrespective of cT category). The main clinical utility was observed in the higher-risk subgroup, in which PET-positive nodal findings were consistently supported by the clinical reference standard (PPV 100%)

The clinical utility of PET/ceCT was strongly stage-dependent. In patients with cT3-4 and/or cN-positive disease, PET-positive nodal findings were consistently supported by the clinical reference standard, yielding a PPV of 100%. In contrast, in cT1-2 cN0 patients, PET/ceCT provided limited additional clinically actionable nodal information. These findings support selective use of PET/ceCT in patients with higher baseline probability of nodal or extra-locoregional disease. The stage-dependent clinical yield of nodal PET/ceCT is shown in Fig. 3.

Baseline clinically confirmed distant metastatic disease was uncommon. Therefore, M-stage results were interpreted mainly as a descriptive analysis of distant disease assessment rather than as a stand-alone sensitivity-driven endpoint. Overall M-stage performance showed sensitivity 57.1%, specificity 55.0%, PPV 7.5%, NPV 95.2%, accuracy 55.2% and kappa 0.030. Table 2 reports PET/ceCT staging performance and stage-dependent clinical yield. Detailed stage-stratified contingency data are provided in Supplementary Table 2 (Table 3).

**Table 2:** Overall diagnostic performance of PET/ceCT in the initial staging cohort

Endpoint	*N*	TP	FP	TN	FN	Sensitivity	Specificity	PPV	NPV	Accuracy	Kappa
Nodal involvement, overall	107	46	4	32	25	64.8%	88.9%	92.0%	56.1%	72.9%	0.469
Distant metastatic disease, overall	116	4	49	60	3	57.1%	55.0%	7.5%	95.2%	55.2%	0.030

**Table 3:** Stage-dependent clinical yield of PET/ceCT nodal assessment

Baseline clinical group	*N*	PET/ceCT nodal-positive findings	Clinically actionable PET/ceCT nodal information	Interpretation
cT1-2 cN0	32	4/32 (12.5%)	0/32 (0.0%)	Limited
cT3-4 and/or cN+	74	45/74 (60.8%)	45/74 (60.8%)	High; PPV for PET-positive nodal findings 100%

### Quantitative PET/ceCT biomarkers and clinicopathological correlations

SUVmax was available in 85/116 patients (73.3%) and ceCT-measured index lesion diameter in 64/116 patients (55.2%). SUVmax correlated positively with Ki-67 (rho = 0.322, *p* = 0.003, q = 0.022), supporting the association between FDG uptake and proliferative activity. SUVmax also differed nominally across molecular subtypes, with the highest values observed in triple-negative/basal-like disease.

ceCT-measured index lesion diameter correlated inversely with ER expression (rho=-0.402, *p* = 0.008, q = 0.039) and PR expression (rho=-0.522, *p* < 0.001, q = 0.006). Additional exploratory correlations were tested between quantitative imaging variables and histology, tumour grade, ER, PR, Ki-67, HER2 status, hormone receptor status, molecular subtype, cT/cN stage, radiological response, pCR, ypT, ypN and lymphovascular invasion. The main statistically and biologically relevant associations are summarized in Table 4, while the complete exploratory correlation matrix and group-comparison results are provided in Supplementary Table 4. Median SUVmax according to molecular subtype is shown in Supplementary Fig. 1 (Tables 5 and 6).

**Table 4:** Spearman correlations between imaging metrics and clinicopathological variables

Imaging metric	Variable	*n*	Statistic	*p* value	q_FDR
SUVmax	ER	49	rho=-0.260	0.071	0.142
SUVmax	PR	49	rho=-0.334	0.019	0.067
SUVmax	Ki-67	82	rho = 0.322	0.003	0.022
SUVmax	Tumour size, cm	10	rho = 0.067	0.854	0.969
SUVmax	Number of positive nodes	11	rho = 0.336	0.313	0.487
SUVmax	cT	21	rho=-0.195	0.398	0.557
SUVmax	cN	21	rho=-0.449	0.041	0.103
ceCT-measured index lesion diameter	ER	42	rho=-0.402	0.008	0.039
ceCT-measured index lesion diameter	PR	42	rho=-0.522	< 0.001	0.006
ceCT-measured index lesion diameter	Ki-67	60	rho = 0.005	0.969	0.969
ceCT-measured index lesion diameter	Tumour size, cm	11	rho = 0.615	0.044	0.103
ceCT-measured index lesion diameter	Number of positive nodes	12	rho = 0.178	0.581	0.740
ceCT-measured index lesion diameter	cT	13	rho = 0.028	0.928	0.969
ceCT-measured index lesion diameter	cN	13	rho=-0.344	0.250	0.438

**Table 5:** Group comparisons for SUVmax and ceCT-measured index lesion diameter

Imaging metric	Grouping variable	*n*	Test	*p* value	q_FDR	Summary
SUVmax	Molecular subtype	85	Kruskal-Wallis	0.015	0.207	Highest median in TNBC/basal-like: 11.90
SUVmax	Grade	20	Kruskal-Wallis	0.160	0.373	No robust difference
SUVmax	HER2 status	82	Mann-Whitney U	0.842	0.842	HER2 + median 6.30; HER2- median 6.50
SUVmax	Hormone receptor status	49	Mann-Whitney U	0.070	0.262	HR+ median 5.90; HR- median 10.90
SUVmax	cN status	21	Mann-Whitney U	0.117	0.328	No robust difference
SUVmax	Baseline risk group	21	Mann-Whitney U	0.276	0.441	No robust difference
SUVmax	pCR status	86	Mann-Whitney U	0.222	0.441	pCR median 7.12; non-pCR median 5.95
ceCT-measured index lesion diameter	Molecular subtype	64	Kruskal-Wallis	0.385	0.489	TNBC/basal-like median 35 mm
ceCT-measured index lesion diameter	Grade	22	Kruskal-Wallis	0.550	0.592	No robust difference
ceCT-measured index lesion diameter	HER2 status	60	Mann-Whitney U	0.059	0.262	HER2- median 32.5 mm; HER2 + median 20.5 mm
ceCT-measured index lesion diameter	Hormone receptor status	42	Mann-Whitney U	0.075	0.262	HR- median 40 mm; HR+ median 24 mm
ceCT-measured index lesion diameter	cN status	13	Mann-Whitney U	0.315	0.441	No robust difference
ceCT-measured index lesion diameter	Baseline risk group	13	Mann-Whitney U	0.315	0.441	No robust difference
ceCT-measured index lesion diameter	pCR status	64	Mann-Whitney U	0.505	0.589	No robust difference

**Table 6:** Restaging performance using longitudinal clinical-imaging adjudication

Analysis	*N*	TP	FP	TN	FN	Sensitivity	Specificity	PPV	NPV	Accuracy	Kappa
Clinical-imaging adjudication	311	219	6	86	0	100%	93.5%	97.3%	100%	98.1%	0.953

### Exploratory PET/ceCT discordance analysis

An exploratory review of PET/ceCT discordance patterns was performed to describe the contribution of integrated metabolic and morphologic assessment. In all 405 matched examinations included in the discordance screen, 165 examinations contained concordant PET-positive/ceCT-positive findings, 9 contained PET-positive findings without a clear morpho-structural CT correlate, 33 contained ceCT morphologic findings without significant FDG uptake, 2 showed mixed PET-only and ceCT-only findings, and 196 showed no relevant PET/ceCT discordance. In the primary staging index examinations (*n* = 101), the corresponding counts were 61, 3, 15, 2 and 20, respectively. Lesion-context counts were non-mutually exclusive. Across all matched examinations, concordant PET-positive/ceCT-positive findings involved breast/local (*n* = 107), nodal (*n* = 99), bone (*n* = 60) and visceral (*n* = 42) contexts. PET-positive findings without a clear CT correlate involved visceral (*n* = 6), bone (*n* = 3), nodal (*n* = 3) and breast/local (*n* = 2) contexts, whereas ceCT morphologic findings without significant FDG uptake involved nodal (*n* = 21), visceral (*n* = 20), bone (*n* = 8) and breast/local (*n* = 4) contexts. This analysis was descriptive and was not intended to quantify the incremental diagnostic value of ceCT over PET alone, because independent blinded PET-only and ceCT-only lesion-by-lesion readings were not performed. Full discordance categories and lesion-context breakdown are reported in Supplementary Table 4.

### Restaging performance and longitudinal relapse assessment

Restaging performance for disease progression was assessed using longitudinal clinical-imaging adjudication. In 311 evaluable examinations, PET/ceCT showed sensitivity 100%, specificity 93.5%, PPV 97.3%, NPV 100%, accuracy 98.1% and kappa 0.953. Examination-level adjudication categories are summarized in Supplementary Tables 5, and a graphical summary is provided in Supplementary Fig. 2.

Among 82 patients with first confirmed PET/ceCT-detected relapse, the first relapse pattern was multiorgan/multisystem in 48 patients (58.5%), visceral-only in 15 (18.3%), nodal/locoregional-only in 9 (11.0%), bone-only in 7 (8.5%) and other-only in 3 (3.7%). Median time to first confirmed PET/ceCT-detected relapse was 24.6 months (IQR 13.0-63.5). Conversion from an initial bone-only or nodal/locoregional-only pattern to visceral disease occurred in 5/16 eligible patients (31.3%). Relapse pattern distribution according to molecular subtype, overall relapse-pattern distribution and conversion analyses are shown in Tables 7, 8 and 9. Additional graphical summaries of first confirmed relapse pattern, relapse pattern by subtype and metastatic evolution are provided in Supplementary Figs. 3–5. Among the 48 patients with first confirmed multiorgan/multisystem relapse, 24 first-relapse examinations could be linked to the exploratory PET/ceCT discordance classification subset; at the first-relapse examination, 14 were concordant PET-positive/ceCT-positive, 9 showed no relevant PET/ceCT discordance and 1 was PET-positive without a clear CT correlate. When all available matched examinations from these 24 patients were considered, PET/ceCT discordance was identified in 4/24 patients (16.7%), and same-examination coexistence of concordant and discordant lesion contexts was documented in 1/24 patients (4.2%). These findings are summarized in Supplementary Table 4.

**Table 7:** First confirmed PET/ceCT-detected relapse pattern by molecular subtype

Molecular subtype	Confirmed relapse, *n*	Bone-only	Visceral-only	Nodal/locoregional-only	Multiorgan/multisystem	Other-only
Luminal B	41	5	10	3	22	1
HER2-positive	16	0	3	4	9	0
Triple-negative/basal-like	12	0	2	1	7	2
Luminal A	10	2	0	1	7	0
Other / not classifiable	3	0	0	0	3	0
Overall	82	7	15	9	48	3

**Table 8:** Overall distribution of first confirmed PET/ceCT-detected relapse patterns

Pattern	*n* / 82	%
Multiorgan/multisystem	48/82	58.5%
Visceral-only	15/82	18.3%
Nodal/locoregional-only	9/82	11.0%
Bone-only	7/82	8.5%
Other-only	3/82	3.7%

**Table 9:** Conversion from bone-only or nodal/locoregional relapse to visceral disease

Molecular subtype	Eligible patients	Conversion to visceral disease	%
Luminal B	8	3	37.5%
HER2-positive	4	1	25.0%
Triple-negative/basal-like	1	0	0%
Luminal A	3	1	33.3%
Overall	16	5	31.3%

## Discussion

In this retrospective single-centre cohort, same-session [18F]FDG PET/ceCT provided clinically relevant but clearly stage-dependent information in breast cancer patients undergoing initial staging. The main baseline finding was not a uniformly high diagnostic accuracy across all endpoints, but rather a selective clinical utility concentrated in patients with cT3-4 and/or cN-positive disease. In this subgroup, PET/ceCT-positive nodal findings were consistently supported by the clinical reference standard, yielding a PPV of 100%. Conversely, PET/ceCT added little clinically actionable nodal information in cT1-2 cN0 patients. These data are consistent with current risk-adapted recommendations and with the broader literature indicating that FDG PET/CT should not be used indiscriminately in early breast cancer, but has higher yield in stage IIB or higher, locally advanced, or clinically high-risk disease [1–3, 13, 14]. The stage-dependent framework illustrated in Figs. 2 and 3 supports the selective use of PET/ceCT in clinically higher-risk disease.

The nodal results deserve a cautious interpretation. Overall nodal sensitivity was moderate, whereas specificity and PPV were high. This pattern supports PET/ceCT as a rule-in rather than rule-out tool for clinically relevant nodal disease. PET/CT cannot replace surgical axillary staging or sentinel node procedures in early-stage disease, because microscopic nodal involvement remains below the spatial and biological detection limits of FDG imaging. However, PET/ceCT may identify clinically meaningful nodal disease outside routine axillary surgical fields, including extra-axillary, supraclavicular, internal mammary or more extensive nodal involvement, with potential implications for systemic treatment planning and radiotherapy fields [6, 13, 14]. False-negative nodal PET/ceCT findings in the higher-risk group may be explained by microscopic or small-volume nodal disease, partial-volume effects, low tumour cellularity or low glycolytic activity, particularly in some luminal or low-proliferative tumours, and anatomical proximity to physiological or inflammatory uptake. Pathologically confirmed nodal disease may also include micrometastatic or limited-volume involvement without a definite metabolic or morphologic abnormality at baseline imaging. These considerations reinforce that PET/ceCT-positive nodal findings are highly informative in high-risk patients, whereas PET/ceCT-negative nodal findings cannot exclude microscopic nodal involvement or replace surgical/pathological nodal staging. Therefore, the strongest baseline message from the present cohort is not that PET/ceCT should be used for all newly diagnosed patients, but that a positive nodal PET/ceCT finding in clinically higher-risk disease is highly informative.

By contrast, baseline M-stage assessment was limited by the low prevalence of clinically confirmed metastatic disease in the staging cohort. Although PET/ceCT is known to detect unsuspected distant metastases in high-risk and locally advanced breast cancer, the small number of confirmed M1 cases in our cohort makes sensitivity, specificity and PPV for baseline M-stage unstable and not suitable as a definitive estimate of metastatic staging accuracy. This is an important limitation, particularly because recent prospective and randomized evidence has shown a stronger effect of PET/CT on detection of stage IV disease in locally advanced breast cancer. In the randomized trial by Dayes et al., PET/CT upstaged 23% of patients to stage IV compared with 11% after conventional staging, and fewer patients in the PET/CT arm received curative-intent combined modality therapy [14]. Our data should therefore be interpreted as supporting stage-dependent clinical yield and high nodal PPV, rather than as challenging the established value of PET/CT for distant staging in appropriately selected high-risk populations.

The present findings also fit with studies suggesting that the threshold for PET/CT use may be influenced not only by anatomical stage but also by tumour biology. Ulaner et al. reported that FDG PET/CT detected unsuspected distant metastases in 15% of patients with newly diagnosed stage IIB triple-negative breast cancer, with significantly worse survival among patients upstaged to stage IV [15]. In a separate cohort of ER-positive/HER2-negative and HER2-positive breast cancer, the same group found unsuspected distant metastases in 14% of stage IIB patients, with higher rates in stage III disease [16]. In our cohort, SUVmax correlated positively with Ki-67 and was highest in triple-negative/basal-like tumours, supporting the link between FDG uptake and proliferative/aggressive biology [2, 7, 13]. The inverse correlations between ceCT-measured index lesion diameter and ER/PR expression are also biologically plausible, but should be considered exploratory because of missing data, retrospective acquisition and multiple testing.

The longitudinal component of the study provides a complementary message. While baseline staging asks whether PET/ceCT should be performed in a newly diagnosed patient, follow-up and restaging address a different clinical question: how PET/ceCT captures relapse phenotype and metastatic evolution over time. In evaluable restaging examinations, PET/ceCT showed very high performance after longitudinal clinical-imaging adjudication. The absence of false-negative examinations in this adjudicated dataset and the high kappa value suggest that integrated PET/ceCT is robust for detecting clinically confirmed relapse or progression in a real-world follow-up setting. However, this estimate should not be interpreted as equivalent to a prospective blinded diagnostic accuracy study, because PET-positive findings not classifiable within the predefined adjudication framework were excluded from performance calculations and clinical follow-up contributed to final adjudication.

The relapse phenotype analysis showed that first confirmed PET/ceCT-detected relapse was most often multiorgan/multisystem or visceral-only. Bone-only and nodal/locoregional-only relapse represented a minority of first relapse patterns, and approximately one third of eligible patients with initial bone-only or nodal/locoregional-only relapse subsequently converted to visceral disease. These findings extend the value of PET/ceCT beyond binary recurrence detection. Serial PET/ceCT may help characterize the trajectory of metastatic breast cancer, distinguishing limited disease phenotypes from early multiorgan dissemination and identifying patients who evolve toward visceral involvement. This point is clinically relevant because metastatic pattern, disease burden and organ involvement influence systemic treatment decisions, local treatment strategies and prognosis. The exploratory discordance review also addressed intra-patient metabolic-morphologic heterogeneity in multiorgan relapse. Among first confirmed multiorgan/multisystem relapse examinations that could be linked to the discordance classification subset, most were concordant PET-positive/ceCT-positive or had no relevant PET/ceCT discordance. Across all available matched examinations from these patients, PET/ceCT discordance was documented in 4/24 evaluable patients and same-examination coexistence of concordant and discordant lesion contexts in 1/24. Therefore, intra-patient heterogeneity was present at least descriptively, but it appeared uncommon in the available screen and should be interpreted cautiously because no independent lesion-by-lesion PET-only and ceCT-only reader study was performed.

The integrated PET/ceCT design is both a strength and a methodological constraint. Same-session PET/ceCT reflects routine clinical practice and allows metabolic and morphologic findings to be interpreted together. This is particularly relevant for bone lesions, small morphologic abnormalities, post-treatment changes and lesions with discordant metabolic and anatomical behaviour. The present study did not show, and was not designed to show, a clear superiority of PET over ceCT or of ceCT over PET. First, PET and ceCT were interpreted as an integrated same-session examination rather than as independent tests. Second, no blinded PET-only and ceCT-only reader arms were performed. Third, the clinical reference standard incorporated integrated PET/ceCT interpretation and longitudinal clinical-imaging information. Therefore, the discordance analysis should be interpreted as a description of complementary metabolic and morphologic information, not as a direct comparison of stand-alone PET versus ceCT lesion detection. FDG uptake and iodinated contrast enhancement also reflect different biological properties: FDG uptake is related mainly to glucose metabolism, cellularity and viable tumour burden, whereas contrast enhancement reflects vascularity, perfusion and blood-tissue barrier properties. These mechanisms may produce modality-specific lesion conspicuity. Small or metabolically indolent lesions, treated lesions and low-proliferative luminal disease may show low FDG uptake despite morphologic abnormality, whereas early marrow, nodal or metabolically active lesions may be FDG-positive before definite CT changes. In metastatic breast cancer, Riedl et al. showed that response assessment by FDG PET/CT using PERCIST was frequently discordant from CE-CT using RECIST and was more strongly associated with progression-free and disease-specific survival [17]. A systematic review also emphasized that direct comparative evidence remains limited, but available data suggest that metabolic response assessment may better capture clinically relevant tumour behaviour than size-based CT criteria alone in metastatic breast cancer [18]. In our study, however, discordant PET/ceCT findings were analysed descriptively and should not be used to claim an independent incremental value of ceCT over PET alone, because PET-only and PET/ceCT readings were not performed as a blinded comparative reader study.

Several limitations must be acknowledged. First, the retrospective single-centre design introduces referral, indication and verification biases. Second, imaging was performed over a long time interval, with different scanner generations and evolving reconstruction protocols; therefore, SUVmax analyses were considered exploratory and interpreted in relation to clinical context rather than as fully standardized quantitative biomarkers. Third, the retrospective design and long study interval limited the completeness of selected variables, particularly ceCT-measured index lesion diameter, tumour grade and selected pathological endpoints in some legacy examinations. This reflected institutional archive migration and heterogeneous historical storage of imaging and clinical documentation. Analyses were therefore restricted to information verifiable in institutional records, and classifications were not forced when documentation was incomplete. This may have reduced statistical power and introduced residual ascertainment bias. Fourth, the reference standard was clinically adjudicated using pathology when available, follow-up imaging, treatment decisions and multidisciplinary assessment. This is pragmatic and clinically realistic, but weaker than systematic histological confirmation for all positive and negative findings. Fifth, baseline M-stage performance was constrained by the low number of clinically confirmed M1 cases. Finally, longitudinal restaging performance may be overestimated because only examinations with adequate clinical-imaging verification were included in diagnostic performance calculations.

Despite these limitations, the study has several strengths. The staging cohort was restricted to one index examination per patient selected according to predefined clinical matching criteria, reducing duplication and indication ambiguity. The longitudinal cohort included a large number of PET/ceCT examinations and allowed patient-level assessment of first confirmed relapse pattern, time to relapse and metastatic evolution. The analysis also separates baseline staging from restaging, avoiding the common problem of mixing distinct clinical questions. Overall, our findings support the selective use of same-session [18F]FDG PET/ceCT in clinically higher-risk breast cancer at baseline and reinforce its value during follow-up for defining relapse phenotype and metastatic evolution. The data do not support routine PET/ceCT use in low-risk cT1-2 cN0 disease, nor do they establish PET/ceCT as a substitute for surgical axillary staging. Rather, they support PET/ceCT as an integrated metabolic-morphologic tool with greatest utility when pre-test probability is high and when longitudinal assessment of disease phenotype is clinically relevant.

## Conclusions

Same-session [18F]FDG PET/contrast-enhanced CT provided clinically useful but stage-dependent information in breast cancer patients undergoing initial staging. Its main baseline value was observed in patients with cT3-4 and/or cN-positive disease, in whom PET/ceCT-positive nodal findings showed high clinical reliability. Conversely, PET/ceCT offered limited actionable information in cT1-2 cN0 patients and should not be considered a substitute for surgical axillary staging. Baseline M-stage assessment in this cohort was constrained by the low prevalence of clinically confirmed metastatic disease and should therefore be interpreted descriptively.

In the longitudinal setting, PET/ceCT showed high performance after clinical-imaging adjudication and allowed characterization of relapse phenotype and metastatic evolution over time. First confirmed PET/ceCT-detected relapse was frequently multiorgan/multisystem or visceral, and conversion from initially bone-only or nodal/locoregional relapse to visceral disease occurred in a relevant subset of patients. These findings support the role of integrated PET/ceCT as a selective staging tool in higher-risk breast cancer and as a clinically useful modality for restaging and longitudinal assessment of metastatic patterns.

## Supplementary Information

Below is the link to the electronic supplementary material.

Supplementary Material 1 Supplementary Fig. 1. Median SUVmax according to molecular subtype. Summary graph of median SUVmax values across molecular subtype groups using the study dataset. The highest median SUVmax was observed in TNBC/basal-like tumours. Group comparison was performed with the Kruskal-Wallis test (p = 0.0148).

Supplementary Material 2 Supplementary Fig. 2. Restaging performance after longitudinal clinical-imaging adjudication. Bar chart showing the main performance metrics of PET/ceCT in the enhanced adjudicated longitudinal subset of 311 examinations. Sensitivity was 100.0%, specificity 93.5%, PPV 97.3%, NPV 100.0%, and accuracy 98.1%. TP = 219, FP = 6, TN = 86, and FN = 0. Cohen's kappa was 0.953.

Supplementary Material 3 Supplementary Fig. 3. First confirmed PET/ceCT-detected relapse pattern. Distribution of the first confirmed PET/ceCT-detected relapse pattern in 82 patients with clinically confirmed PET-positive relapse. Multiorgan/multisystem relapse was the most frequent pattern (48/82, 58.5%), followed by visceral-only relapse (15/82, 18.3%), nodal/locoregional-only relapse (9/82, 11.0%), bone-only relapse (7/82, 8.5%), and other-only relapse (3/82, 3.7%).

Supplementary Material 4 Supplementary Fig. 4. First confirmed relapse pattern according to molecular subtype. Stacked bar chart showing the distribution of the first confirmed PET/ceCT-detected relapse pattern across subtype groups. The graph summarizes patients with clinically confirmed PET-positive relapse and highlights the predominance of multiorgan/multisystem relapse across subtypes.

Supplementary Material 5 Supplementary Fig. 5. Metastatic evolution from bone-only or nodal/locoregional-only relapse. Among 16 eligible patients with an initial confirmed bone-only or nodal/locoregional-only relapse pattern, 5 patients (31.3%) subsequently developed visceral disease on follow-up.

Supplementary Material 6

## Data Availability

The datasets generated and analyzed during the current study are not publicly available due to institutional and privacy restrictions but are available from the corresponding author on reasonable request.

## Code availability

Custom code/scripts used for image processing and statistical analyses are available from the corresponding author on reasonable request.
