# Supplementary material for: Stage-dependent performance and molecular subtype-specific relapse patterns on same-session [18F]FDG PET/contrast-enhanced CT in breast cancer
Source: Eur J Nucl Med Mol Imaging. 2026 Aug 7;53(13):8189–202. doi: 10.1007/s00259-026-08077-w (PMC13727870; doi:10.1007/s00259-026-08077-w)
Supplement: Supplementary file 1 [file 259_2026_8077_MOESM6_ESM.docx]

**Supplementary Table 1**

**Clinical, pathological and imaging characteristics of the primary staging cohort**

Available/evaluable cases are reported for each variable. Percentages are calculated using the variable-specific denominator.

| **Variable** | **Result** |
| --- | --- |
| Patients | 116 |
| Women / men | 115 / 1 |
| Age, years | 48 (IQR 42-60) |
| Same-session PET/ceCT with intravenous contrast | 108/116 (93.1%) |
| Baseline clinical stage evaluable | 106/116 |
| cT1-2 cN0 | 32/106 (30.2%) |
| cT3-4 cN0 | 9/106 (8.5%) |
| cN-positive, any cT | 65/106 (61.3%) |
| cT3-4 and/or cN+ | 74/106 (69.8%) |
| SUVmax available for quantitative/subtype analysis | 85/116 (73.3%) |
| ceCT-measured index lesion diameter available | 64/116 (55.2%) |
| Histology evaluable | 108 |
| Invasive ductal carcinoma / NST | 94/108 (87.0%) |
| Invasive lobular carcinoma | 2/108 (1.9%) |
| Other / special / not otherwise specified | 12/108 (11.1%) |
| Tumour grade evaluable | 39 |
| G1 | 5/39 (12.8%) |
| G2 | 17/39 (43.6%) |
| G3 | 17/39 (43.6%) |
| ER evaluable | 105 |
| ER-positive | 80/105 (76.2%) |
| ER-negative | 25/105 (23.8%) |
| PR evaluable | 105 |
| PR-positive | 69/105 (65.7%) |
| PR-negative | 36/105 (34.3%) |
| HER2 evaluable | 105 |
| HER2-positive | 33/105 (31.4%) |
| HER2-negative | 72/105 (68.6%) |
| Ki-67 evaluable | 105 |
| Ki-67 | 35% (IQR 20-55) |
| Molecular subtype evaluable | 113 |
| HR+/HER2- | 59/113 (52.2%) |
| HER2-positive overall | 33/113 (29.2%) |
| Triple-negative/basal-like | 18/113 (15.9%) |
| Other / not classifiable | 3/113 (2.7%) |
| cT evaluable | 95 |
| cT1 | 10/95 (10.5%) |
| cT2 | 61/95 (64.2%) |
| cT3 | 13/95 (13.7%) |
| cT4 | 11/95 (11.6%) |
| cN evaluable | 96 |
| cN0 | 31/96 (32.3%) |
| cN1 | 52/96 (54.2%) |
| cN2 | 11/96 (11.5%) |
| cN3 | 2/96 (2.1%) |
| pCR evaluable | 90 |
| pCR | 35/90 (38.9%) |
| Non-pCR | 55/90 (61.1%) |
| ypN evaluable | 90 |
| ypN-positive residual disease | 34/90 (37.8%) |
| ypN0 | 56/90 (62.2%) |

**Supplementary Table 2**

**PET/ceCT diagnostic performance and 2x2 contingency matrices**

Diagnostic metrics were calculated against the clinically adjudicated reference standard. Stage-dependent subgroup analyses are presented as descriptive clinical-yield analyses when event numbers were sparse.

**A. Overall initial staging diagnostic performance**

| **Endpoint** | **N** | **TP** | **FP** | **TN** | **FN** | **Sensitivity** | **Specificity** | **PPV** | **NPV** | **Accuracy** | **Kappa** |
| --- | --- | --- | --- | --- | --- | --- | --- | --- | --- | --- | --- |
| Nodal staging | 107 | 46 | 4 | 32 | 25 | 64.8% | 88.9% | 92.0% | 56.1% | 72.9% | 0.469 |
| M-stage | 116 | 4 | 49 | 60 | 3 | 57.1% | 55.0% | 7.5% | 95.2% | 55.2% | 0.030 |

**B. Stage-dependent clinical yield and contingency data**

| **Baseline clinical group** | **Endpoint** | **N** | **TP** | **FP** | **TN** | **FN** | **Sensitivity** | **Specificity** | **PPV** | **NPV** | **Accuracy** | **Kappa** | **Interpretation** |
| --- | --- | --- | --- | --- | --- | --- | --- | --- | --- | --- | --- | --- | --- |
| cT1-2 cN0 | Nodal staging | 32 | 0 | 4 | 28 | 0 | Not applicable | 87.5% | 0.0% | 100.0% | 87.5% | 0 | Limited additional actionable yield |
| cT3-4 and/or cN+ | Nodal staging | 74 | 45 | 0 | 4 | 25 | 64.3% | 100.0% | 100.0% | 13.8% | 66.2% | 0.163 | Higher actionable yield |
| cT1-2 cN0 | M-stage (descriptive subgroup analysis) | 32 | 0 | 16 | 16 | 0 | Not applicable | 50.0% | 0.0% | 100.0% | 50.0% | 0 | Descriptive only |
| cT3-4 and/or cN+ | M-stage (descriptive subgroup analysis) | 74 | 0 | 31 | 43 | 0 | Not applicable | 58.1% | 0.0% | 100.0% | 58.1% | 0 | Descriptive only |

M-stage subgroup estimates should be interpreted descriptively because clinically confirmed metastatic events were sparse and were not evenly distributed across baseline clinical groups.

**C. Restaging/follow-up performance**

| **Analysis framework** | **N exams** | **TP** | **FP** | **TN** | **FN** | **Sensitivity** | **Specificity** | **PPV** | **NPV** | **Accuracy** | **Kappa** |
| --- | --- | --- | --- | --- | --- | --- | --- | --- | --- | --- | --- |
| Initial examination-level screen (sensitivity analysis) | 429 | 88 | 255 | 68 | 18 | 83.0% | 21.1% | 25.7% | 79.1% | 36.4% | 0.023 |
| Longitudinal clinical-imaging adjudication | 311 | 219 | 6 | 86 | 0 | 100.0% | 93.5% | 97.3% | 100.0% | 98.1% | 0.953 |

**Supplementary Table 3**

**Exploratory correlations between PET/ceCT biomarkers and clinical-pathological variables**

SUVmax and ceCT-measured index lesion diameter were analysed as exploratory imaging biomarkers. P values were adjusted using the Benjamini-Hochberg false discovery rate procedure.

**A. Spearman correlations**

| **Metric** | **Variable** | **n** | **rho** | **p value** | **q_FDR** |
| --- | --- | --- | --- | --- | --- |
| SUVmax | ER | 49 | -0.260 | 0.071 | 0.142 |
| SUVmax | PR | 49 | -0.334 | 0.019 | 0.067 |
| SUVmax | Ki-67 | 82 | 0.322 | 0.003 | 0.022 |
| SUVmax | Pathological tumour size, cm | 10 | 0.067 | 0.854 | 0.969 |
| SUVmax | Number of positive nodes | 11 | 0.336 | 0.313 | 0.486 |
| SUVmax | cT | 21 | -0.195 | 0.398 | 0.557 |
| SUVmax | cN | 21 | -0.449 | 0.041 | 0.103 |
| ceCT-measured index lesion diameter | ER | 42 | -0.402 | 0.008 | 0.039 |
| ceCT-measured index lesion diameter | PR | 42 | -0.522 | <0.001 | 0.005 |
| ceCT-measured index lesion diameter | Ki-67 | 60 | 0.005 | 0.969 | 0.969 |
| ceCT-measured index lesion diameter | Pathological tumour size, cm | 11 | 0.615 | 0.044 | 0.103 |
| ceCT-measured index lesion diameter | Number of positive nodes | 12 | 0.177 | 0.581 | 0.740 |
| ceCT-measured index lesion diameter | cT | 13 | 0.028 | 0.928 | 0.969 |
| ceCT-measured index lesion diameter | cN | 13 | -0.344 | 0.250 | 0.438 |

**B. Group comparisons**

| **Metric** | **Grouping variable** | **n** | **Groups** | **Test** | **p value** | **q_FDR** | **Summary** |
| --- | --- | --- | --- | --- | --- | --- | --- |
| SUVmax | Molecular subtype | 85 | 4 | Kruskal-Wallis | 0.015 | 0.207 | Highest median value in TNBC/basal-like tumours (11.90); lower values in HER2-positive, Luminal B and Luminal A groups |
| SUVmax | Grade | 20 | 5 | Kruskal-Wallis | 0.160 | 0.373 | No robust difference across grade categories |
| SUVmax | HER2 status | 82 | 2 | Mann-Whitney U | 0.842 | 0.842 | HER2+: median 6.30 (n=29); HER2-: median 6.50 (n=53) |
| SUVmax | Hormone receptor status | 49 | 2 | Mann-Whitney U | 0.070 | 0.262 | HR+: median 5.90 (n=39); HR-: median 10.90 (n=10) |
| SUVmax | cN status | 21 | 2 | Mann-Whitney U | 0.117 | 0.328 | cN+: median 5.30 (n=14); cN0: median 9.70 (n=7) |
| SUVmax | Baseline clinical risk group | 21 | 2 | Mann-Whitney U | 0.276 | 0.441 | cT1-2 cN0: median 8.52 (n=6); cT3-4 and/or cN+: median 5.50 (n=15) |
| SUVmax | pCR status | 86 | 2 | Mann-Whitney U | 0.222 | 0.441 | non-pCR: median 5.95 (n=46); pCR: median 7.12 (n=40) |
| ceCT-measured index lesion diameter | Molecular subtype | 64 | 4 | Kruskal-Wallis | 0.385 | 0.489 | Largest median diameter in TNBC/basal-like tumours (35.00 mm); group difference not statistically robust |
| ceCT-measured index lesion diameter | Grade | 22 | 5 | Kruskal-Wallis | 0.550 | 0.592 | No robust difference across grade categories |
| ceCT-measured index lesion diameter | HER2 status | 60 | 2 | Mann-Whitney U | 0.059 | 0.262 | HER2+: median 20.50 (n=20); HER2-: median 32.50 (n=40) |
| ceCT-measured index lesion diameter | Hormone receptor status | 42 | 2 | Mann-Whitney U | 0.075 | 0.262 | HR+: median 24.00 (n=36); HR-: median 40.00 (n=6) |
| ceCT-measured index lesion diameter | cN status | 13 | 2 | Mann-Whitney U | 0.315 | 0.441 | cN+: median 20.00 (n=9); cN0: median 32.50 (n=4) |
| ceCT-measured index lesion diameter | Baseline clinical risk group | 13 | 2 | Mann-Whitney U | 0.315 | 0.441 | cT1-2 cN0: median 32.50 (n=4); cT3-4 and/or cN+: median 20.00 (n=9) |
| ceCT-measured index lesion diameter | pCR status | 64 | 2 | Mann-Whitney U | 0.505 | 0.589 | non-pCR: median 28.00 (n=37); pCR: median 30.00 (n=27) |

**Supplementary Table 4**

**Exploratory analysis of discordant PET and contrast-enhanced CT findings**

This descriptive analysis reports PET/ceCT discordance patterns observed during integrated image review. It should not be interpreted as a blinded reader-study quantifying the incremental diagnostic value of ceCT over PET alone.

**A. PET/ceCT discordance summary**

| **Cohort** | **N examinations** | **Concordant PET+/ceCT+ findings** | **PET-positive without clear CT correlate** | **ceCT morphologic finding without significant FDG uptake** | **Mixed PET-only and ceCT-only findings** | **No relevant PET/ceCT discordance** |
| --- | --- | --- | --- | --- | --- | --- |
| Primary staging index examinations | 101 | 61 | 3 | 15 | 2 | 20 |
| All matched examinations | 405 | 165 | 9 | 33 | 2 | 196 |
| Matched restaging/follow-up examinations | 258 | 79 | 4 | 14 | 0 | 161 |

**B. Operational definitions**

| **Discordance category** | **Operational definition** | **Clinical use in this study** |
| --- | --- | --- |
| Concordant PET-positive/ceCT-positive | Metabolic abnormality with corresponding morphologic/contrast-enhanced CT finding | Hybrid finding; supports integrated PET/ceCT interpretation |
| PET-positive without clear CT correlate | Focal FDG uptake without a definite morphologic correlate | Requires clinical/imaging follow-up, lesion-specific context and potential further workup |
| ceCT morphologic finding without significant FDG uptake | Morphologic or enhancing abnormality on ceCT with low/absent FDG uptake | May support lesion detection/measurement and highlights low-FDG or treated disease patterns |
| Mixed PET-only and ceCT-only findings | Coexistence of both discordance types in the same examination | Requires case-by-case integrated assessment |
| No relevant PET/ceCT discordance | No clear discordance pattern in the reviewed examination | No discordance-specific interpretation required |

**C. Lesion-context distribution of exploratory PET/ceCT findings**

| **PET/ceCT pattern** | **Breast/local** | **Nodal** | **Bone** | **Visceral** | **Interpretation** |
| --- | --- | --- | --- | --- | --- |
| Concordant PET-positive/ceCT-positive | 107 | 99 | 60 | 42 | Metabolic and morphologic/contrast-enhanced CT findings were both present in the same anatomical context |
| PET-positive without clear CT correlate | 2 | 3 | 3 | 6 | FDG uptake was described without a definite corresponding morphologic correlate |
| ceCT morphologic finding without significant FDG uptake | 4 | 21 | 8 | 20 | Morphologic/contrast-enhanced CT abnormality was described with low or absent FDG uptake |

Site-context categories are not mutually exclusive because some examinations involved more than one anatomical compartment.

**D. Intra-patient metabolic-morphologic heterogeneity in first confirmed multiorgan/multisystem relapse**

| **Assessment** | **Result** | **Interpretation** |
| --- | --- | --- |
| Patients with first confirmed multiorgan/multisystem relapse | 48 | Overall patient-level multiorgan/multisystem relapse group |
| First-relapse examinations evaluable in the discordance classification subset | 24/48 | Subset with matched first-relapse examination available for exploratory PET/ceCT discordance classification |
| First-relapse examination: concordant PET-positive/ceCT-positive | 14/24 | Both metabolic and morphologic findings were present in the relevant lesion contexts |
| First-relapse examination: no relevant PET/ceCT discordance | 9/24 | No discordance-specific pattern was documented in the first-relapse examination |
| First-relapse examination: PET-positive without clear CT correlate | 1/24 | Metabolic abnormality without a definite CT correlate was documented at first relapse |
| First-relapse examination: ceCT morphologic finding without significant FDG uptake | 0/24 | No first-relapse examination in this subset was classified in this category |
| First-relapse examination: mixed PET-only and ceCT-only findings | 0/24 | No first-relapse examination in this subset was classified as mixed discordance |
| Any PET/ceCT discordance across available matched examinations from these patients | 4/24 (16.7%) | At least one discordant PET/ceCT pattern occurred during available longitudinal assessment |
| Same-examination coexistence of concordant and discordant lesion contexts | 1/24 (4.2%) | Descriptive evidence of intra-patient metabolic-morphologic heterogeneity |

This analysis was descriptive and was not designed to provide exhaustive lesion-by-lesion PET-only versus ceCT-only quantification.

**Supplementary Table 5**

**Longitudinal relapse adjudication and metastatic evolution**

This table summarizes the longitudinal clinical-imaging adjudication framework at examination and patient level. PET-positive findings not classifiable within the predefined adjudication framework were not included in adjudicated diagnostic-performance calculations.

**A. Examination-level adjudication categories**

| **Adjudication category** | **N examinations** | **Percentage of all examinations** | **Use in performance analysis** |
| --- | --- | --- | --- |
| Confirmed PET-positive finding with treatment response or disappearance on follow-up | 113 | 26.3% | Included as true positive when clinically consistent |
| Confirmed PET-positive finding with progression/new disease on follow-up | 35 | 8.2% | Included as true positive |
| Confirmed PET-positive finding with persistent/stable abnormality in a clinically consistent context | 3 | 0.7% | Included as true positive when clinically consistent |
| Confirmed PET-positive finding by concordant clinical-imaging records | 68 | 15.9% | Included as true positive when clinically consistent |
| PET-negative examination | 86 | 20.0% | Included as true negative when no relapse/progression was confirmed |
| PET-positive finding not classifiable within the predefined adjudication framework | 118 | 27.5% | Not included in adjudicated diagnostic-performance calculations |
| PET-positive finding not confirmed despite later follow-up | 6 | 1.4% | Included as false positive in adjudicated performance calculations |

**B. Patient-level first relapse adjudication**

| **Patient-level first relapse category** | **N patients** | **Percentage of longitudinal cohort** | **Interpretation** |
| --- | --- | --- | --- |
| Confirmed PET/ceCT-positive relapse | 82 | 46.6% | Used for first confirmed relapse-pattern analysis |
| PET-positive finding not classifiable for first-relapse analysis | 72 | 40.9% | Not included in confirmed first-relapse pattern denominator |
| No PET/ceCT-positive relapse | 22 | 12.5% | No confirmed PET-positive relapse during available follow-up |
| Total | 176 | 100.0% | Longitudinal cohort |

**C. First confirmed PET/ceCT-detected relapse pattern**

| **First confirmed PET/ceCT-detected relapse pattern** | **N patients** | **Percentage of confirmed relapses** |
| --- | --- | --- |
| Multiorgan/multisystem | 48 | 58.5% |
| Visceral-only | 15 | 18.3% |
| Nodal/locoregional-only | 9 | 11.0% |
| Bone-only | 7 | 8.5% |
| Other-only | 3 | 3.7% |
| Total | 82 | 100.0% |

**D. Metastatic evolution**

| **Initial confirmed relapse pattern** | **Eligible patients** | **Converted to visceral disease** | **Percentage** |
| --- | --- | --- | --- |
| Bone-only or nodal/locoregional-only | 16 | 5 | 31.3% |
